# Acyl-Lipid Δ^6^-Desaturase May Act as a First FAD in Cyanobacteria

**DOI:** 10.3390/biom12121795

**Published:** 2022-12-01

**Authors:** Alexander Y. Starikov, Roman A. Sidorov, Sergei V. Goriainov, Dmitry A. Los

**Affiliations:** 1 K.A. Timiryazev Institute of Plant Physiology, Russian Academy of Sciences, Botanicheskaya Street 25, 127276 Moscow, Russia; 2 Laboratory of High-Resolution Mass Spectrometry and NMR Spectroscopy of the Scientific and Educational Center, Peoples’ Friendship University of Russia (RUDN University), Miklukho-Maklaya Street, Build. 6, 117198 Moscow, Russia

**Keywords:** *Synechococcus*, *Synechocystis*, cyanobacteria, desaturase, fatty acids

## Abstract

Fatty acid desaturases (FADs) play important roles in various metabolic and adaptive pathways in all living organisms. They represent a superfamily of oxygenases that introduce double bonds into the acyl chains of fatty acids (FAs). These enzymes are highly specific to the length of the carbon chain, position of double bonds formation, etc. The modes by which FADs “count” the position of the double bond formation may differ. In cyanobacteria, the first double bond is formed between 9th and 10th carbons (position Δ^9^), counting from the carboxylic end of an FA. Other FADs that produce polyunsaturated FAs may introduce double bonds counting from the carboxyl (Δ) or methyl (ω) terminus, or from a pre-existing double bond towards carboxyl or methyl terminus of an FA chain. Here, we expressed the *desD* gene for the Δ6-FAD from *Synechocystis* sp. PCC 6803 in *Synechococcus elongatus* PCC 7942 (which is capable of synthesizing only monoenoic FAs desaturated mainly at position Δ^9^) and observed the appearance of unusual monoenoic FAs desaturated at position Δ^6^, as well as Δ^6,9^ dienoic FAs. Exogenously added *cis*-10-heptadecenoic acid (17:1Δ^10^) was converted into *cis*-6,10-heptadecadienoic (17:2Δ^6,10^). These data demonstrate the ability of Δ6-FAD to introduce the first double bond into the unsaturated substrates and suggests that it “counts” from the carboxyl end, irrespective of the absence or presence of a previous double bond in an FA chain.

## 1. Introduction

Various representatives of cyanobacteria, microalgae and plants are capable of synthesizing polyunsaturated fatty acids (PUFAs), whose important role in maintaining human health has been shown in many studies. The key enzymes in PUFA synthesis are fatty acid desaturases (FADs)—the oxygenases that remove two hydrogen atoms from an acyl residue, thereby catalyzing the formation of a *cis* double bond in a carbon chain.

Among PUFAs, gamma-linolenic acid (γ-linolenic acid; GLA; 18:3Δ^6,9,12^) belongs to the ω6 family fatty acids (FAs) and appears primarily in some plant oils (evening primrose, blackcurrant, borage, or some other plant oils) and cyanobacterial filamentous *Arthrospira* (*Spirulina*) species. Three fatty acid desaturases (FADs) participate in the synthesis of GLA: (a) Δ9-FAD produces monounsaturated oleic acid (18:1Δ^9^) from saturated stearic acid; (b) Δ12-FAD produces di-unsaturated linoleic acid (18:2Δ^9,12^) from oleic acid; and (c) Δ6-FAD—the key enzyme in the synthesis of GLA—introduces the third double bond at the Δ^6^ position, resulting in tri-unsaturated 18:3Δ^6,9,12^.

Plant microsomal Δ6-FADs are known as front-end desaturases that catalyze the introduction of a double bond between preexisting Δ^9^ double bonds and the carboxyl-group (i.e., the front or Δ-end) of the FA molecule [1]. Plant Δ6-FADs differ from other characterized microsomal FADs in that they have a cytochrome *b*_5_ domain fused to the *N*-terminus of a FAD [2]. The *N*-terminal cytochrome *b*_5_ domain was also found in the vertebrate *Caenorhabditis elegans* homologue of the Δ6-FAD [3].

In cyanobacteria, Δ6-FADs are represented by the DesD family of acyl-lipid desaturases [4]. The corresponding genes have been cloned from *Synechocystis* sp. strain PCC 6803 (*desD* or *sll0262* [5]) and *Arthrospira* (*Spirulina*) *platensis* [6]. It was postulated that in cyanobacteria, as in other organisms, the first double bond is formed at the position Δ^9^ (counting from the carboxylic end of a FA) [7]. Other FADs that produce PUFAs may introduce double bonds between the specific carbon atoms counting from the carboxyl (Δ) or methyl (ω) terminus of the FA chain. Alternatively, a subsequent double bond may be introduced three carbons (3C) apart from the pre-existing double bond towards carboxyl or methyl terminus of an FA chain [8].

Previously, we demonstrated that cyanobacterial Δ12-FADs “count” three C atoms toward the methyl end from an existing double bond in the monoenoic precursors [9].

The counting mode of acyl-lipid Δ6-desaturases have never been studied in detail. Earlier studies could not clarify whether the Δ6-FAD can count the carbon number from the carboxyl terminus or from the double bond at the Δ^9^ position [7]. Here, we expressed the *desD* gene of *Synechocystis* sp. PCC 6803, encoding the acyl-lipid Δ6-desaturase in *Synechococcus elongatus* PCC 7942 (which is capable of synthesizing only monoenoic FAs desaturated at position Δ^9^ or Δ^11^) in order to determine the position of the newly appeared double bond in the fatty acyl chains and to reveal the counting mode of this Δ6-FAD.

## 2. Materials and Methods

### 2.1. Cyanobacterial and Bacterial Strains

During the experiment, strains of the model cyanobacterium *Synechococcus elongatus* PCC 7942 and *Synechocystis* strain sp. PCC 6803 substrain GT were used. The cyanobacterial strains were obtained from the Collection of microalgae and cyanobacteria IPPAS (K.A. Timiryazev Institute of Plant Physiology RAS, Moscow, Russia). Both strains were maintained on a solid BG-11 medium [10] that contained 1.2% agar. The experimental growing conditions were as follows: BG-11 liquid medium with the addition of HEPES-NaOH pH 7.5 in a volume of 250 mL under constant illumination of 50 µE m^−2^ s^−1^ at a temperature of 33 °C. In the experiments with exogenic FAs, the *S. elongatus* strains were grown for 24 h in a liquid BG-11 medium supplemented with potassium salt of heptadecenoic acid, *cis*-17:1**Δ**^10^, at a final concentration of 0.1 mM. To obtain the potassium salt, an equimolar amount of 1 M KOH in 80% ethanol was added to a portion of heptadecenoic acid. Then, the alcohol was evaporated and brought to the initial concentration of 0.1 M. An excess of KOH was neutralized by the buffer capacity of the cultivation medium. The cultures were sparged with sterile air enriched with CO_2_ (1.5%). Planting and cultivation were carried out aseptically, and the samples were withdrawn and fixed in the middle of the exponential growth stage (OD_750nm_ ~ 1.0).

### 2.2. Genetic Constructions

The *desD* gene (*sll0262*) was amplified from the genomic DNA of *Synechocystis* sp. PCC 6803 [11] with primers that contained the specific restriction sites: *sll0262*_*Eco*RI 5′-CTATTTAAATGAATTCATGCTAACAG and *sll0262*_*Bam*HI 5′-CAATCCCAAGGATCCGTCACGATG. Phusion High-Fidelity DNA Polymerase (New England Biolabs, Ipswich, MA, USA) was used for PCR. The amplified DNA fragments were digested with the appropriate restriction endonucleases (*Eco*R I and *Bam*H I), purified using the Cleanup Standard kit (Evrogen, Moscow, Russia) and ligated with the linearized pTrc99A vector (Pharmacea, Uppsala, Sweden; https://www.addgene.org/vector-database/4402 (accessed on 3 November 2022)). This plasmid was propagated in an *E. coli* strain XL-1 (Stratagene, La Jolla, CA, USA) and used as a template for further cloning. The DNA fragment containing the *desD* gene under control of the constitutive Trc promoter was amplified using *Xma* I containing primers (5′-ATTGACCCGGGTTGACAGCTTATC and 5′-5′-CATCCCGGGAAACAGCCAAG) digested with *Xma* I, and inserted into the unique *Xma* I site of pAM1303 [11] (https://www.addgene.org/40243 (accessed on 30 November 2022)). The resulting plasmid, pAM-*desD*, was used to transform *S. elongatus* PCC 7942 by homologous recombination [12]. The selection of transformants was carried out on a solid BG-11 medium containing spectinomycin at a final concentration of 30 µg mL^−1^.

### 2.3. FA Analysis

#### 2.3.1. Synthesis of Fatty Acid Methyl Esters (FAMEs)

Wild-type and transformant (pAM-*desD*) cells of *S. elongatus* PCC 7942 were used to analyze the FA composition. Cells (100 µg) were resuspended in 1 mL of 80% alcohol solution of 1 M KOH and incubated at 65 °C for 1 h. The samples were then washed twice with 500 µL of *n*-hexane to remove an unsaponifiable fraction of FAs. The excess KOH was neutralized by 50 µL of 20% sulfuric acid. Free FAs were extracted from the samples with *n*-hexane (300 µL) and evaporated to dryness. A total of 200 µL of 1% sulfuric acid in methanol was added to the dried pellet. FA methylation was carried out for 30 min at 55 °C. FAMEs were extracted with hexane in a volume of 200 µL.

#### 2.3.2. Pre-Concentration of Minor FA

Free FAs obtained from cultures grown in the presence of heptadecenoic acid were separated using high performance liquid chromatography. FAMEs were separated depending on their equivalent carbon number (ECN) under conditions of an isocratic acetonitrile system (Shimadzu LC20, diode detector SPD20MA, detection wavelength 205 nm, column Zorbax C-18 (250 mm × 4.6 mm, 5 mm). The resulting peaks were collected by a fraction collector and evaluated by the GC-MS method. To reliably determine the double bond in the obtained compounds, the conversion of methyl esters to 3-pyridylcarbinol esters was carried out.

#### 2.3.3. 3-Pyridylcarbinol Esters Synthesis

To obtain 3-pyridylcarbinol derivatives, FAMEs were converted into free FAs by alkaline hydrolysis and dried under argon flow. Then, 200 µL of oxalyl chloride was added, and samples were incubated at 50 °C for 1 h. The excess reagent was distilled off with argon. A total of 30 µL of 20% 3-hydroxymethylpyridine in acetonitrile was added to the resulting acid chlorides and incubated at 100 °C for 2–3 min. The sample volume was adjusted to 200 µL and GC-MS analysis was performed.

### 2.4. GC/MS Parameters

The composition of FAMEs, as well as their 3-pyridylcarbinol derivatives, were analyzed by GC-MS on Shimadzu-2010 Plus GC with a quadrupole mass-detector Shimadzu- 2020QP fitted with a 60-m capillary column HP-88 (inner diameter 0.25 mm, thickness of stationary phase—(88%-cyanopropyl)aryl-polysyloxane)—250 μm). The prepared FA methyl esters were separated under the following conditions: carrier gas Helium at 1 mL min^−1^ and sample volume 1 μL (ca. 10 μg FAMEs). Splitless injection was used and the evaporator temperature was 260 °C. The oven temperature program was as follows: 1 min hold at 120 °C, from 120 °C to 175 °C at 10 °C min^−1^ (10 min hold at this temperature), to 210 °C at 5 °C min^−1^ (5 min hold at this temperature), to 230 °C at 5 °C min^−1^ and 20 min hold at 230 °C. The operational temperature of the mass-detector was set to 200 °C, and the ionization energy to 70 eV.

The prepared 3-pyridylcarbinol esters were separated under the following conditions: carrier gas Helium at 1.5 mL min^−1^ and sample volume 1 μL (ca. 10 μg FAMEs). Splitless injection was used and the evaporator temperature was 260 °C. The oven temperature program was as follows: 90 min hold at 240 °C. The operational temperature of the mass-detector was set to 200 °C, and the ionization energy to 70 eV.

## 3. Results

### 3.1. Expression of the desD Gene in S. elongatus PCC 7942

**Figure 1:**
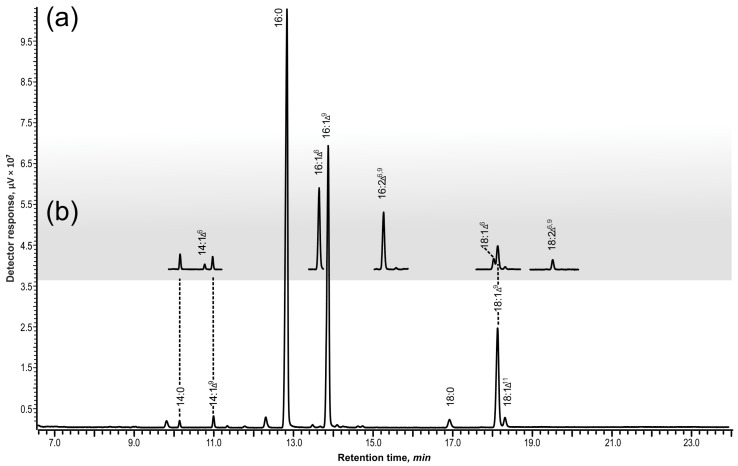
Separation of fatty acids methyl esters obtained from total lipids of strain *Synechococcus elongatus* PCC 7942 by variants (total ion chromatograms): (**a**) wild type cells; (**b**) cells expressed the *desD* (*sll026*) gene (grey plate) and, supposedly, desaturated FAs at position Δ^6^.

The *desD* (*sll0262*) gene is a single gene that encodes the Δ6-FAD in the genome of the model cyanobacterium *Synechocystis* strain sp. PCC 6803 [5]. The *desD* gene was expressed in *S. elongatus* PCC 7942, which possesses only one gene for FADs, *desC* encoding Δ9-FAD, and produces only monounsaturated palmitoleic (16:1Δ^9^) and oleic (18:1Δ^9^) acids, as well as a small amount of *cis*-vaccenic acid (18:1Δ^11^) (Figure 1, Table 1), in which the double bond is formed during 16:1 elongation without participation of any FAD [13].

In addition to these regular FAs, the transgenic *Synechococcus* cells that expressed Δ6-FAD produced unusual FAs, supposedly, monounsaturated at position Δ^6^ (14:1Δ^6^, 16:1Δ^6^, and 18:1Δ^6^), as well as dienoic 14:2Δ^6,9^, 16:2Δ^6,9^ and 18:2Δ^6,9^ (Figure 1, Table 1).

### 3.2. Determination of the Position of Double Bonds in FAs

*S. elongatus* transformant expressing the *desD* (*sll0262*) gene, which was grown in presence of potassium salt of *cis*-10-heptadecenoic acid (17:1**Δ**^10^), displayed the additional dienoic FA, 17:2Δ^10,X^. The latter, supposedly, represented the Δ^6,10^ dienoic product of the corresponding monounsaturated substrate. An amount of heptadecenoic acid in samples was low, and the heptadecadienoic derivative was poorly separated from the stearate. To identify this molecule and to detail epy chromatographic and mass-spectrometry characteristic of other minor UFAs, we separated their methyl esters by equivalent carbon number (ECN).

Fractions of FAMEs separated by their ECN values were obtained with semi-preparative reversed-phase HPLC equipped with a refractive index detector. A fraction corresponding to the ECN value to C12 contained 14:1 and 16:2 FAs. A peak with an ECN value of C14 contained 14:0 and 16:1 acids. Between these two peaks, a minor peak with an ECN value of 13 was detected, which was mainly comprised of heptadecadienoic acid.

Chromatographic properties of FAMEs, as well as mass-spectral data of their 3-pyridylcarbinyl esters, are presented in Table 2.

### 3.3. Determination of the Position of the Second Double Bond in Fas

**Figure 2:**
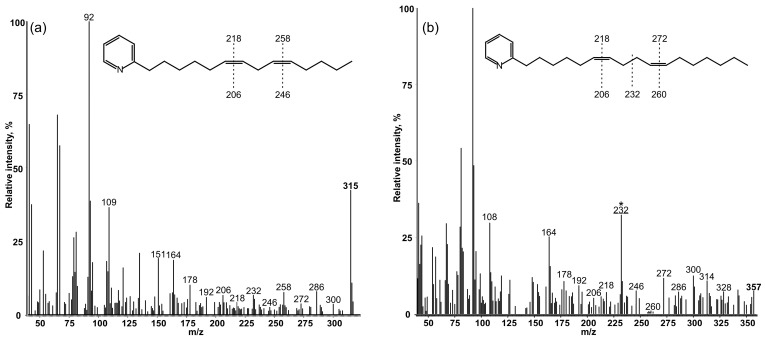
Mass-spectra 3-pyridylcarbinyl derivatives of (**a**) *cis*,*cis*-6,9-tetradecadienoic and (**b**) *cis*,*cis*-6,10-heptadecadienoic acids, which were recorded after pre-concentration of FAMEs from the total lipids of *Synechococcus* cells that expressed the *desD* gene and were grown in the presence of the potassium salt of *cis*-17:1Δ^10^.

To precisely locate double bond positions in these FA chains, we used mass-spectrometry of their 3-pyridylcarbinyl derivatives (Figure 2). The molecular ion *m*/*z* = 315 (Figure 2a) belongs to the 3-pyridylcarbinyl derivative of tetradecadienoic acid. Its molecular ion peak was determined at *m*/*z* = 315. It is typical in that it has prominent ions at *m*/*z* = 92, 108, 151 and 164, which are all fragments of the pyridine ring (https://www.lipidmaps.org/resources/lipidweb/lipidweb_html/ms/pyrcarb.htm (accessed on 30 November 2022)), and ions with m/z up to 315 are formed by fragmentation of an acyl chain.

All these gaps clearly demonstrate the location of the double bonds at positions Δ^6^ and Δ^9^. The 12 amu gaps observed at *m*/*z* = 206–218 and *m*/*z* = 246–258 correlate well with this conclusion (Figure 2a).

The transformant that expressed the *desD* gene converted exogenically added *cis*-Δ10-heptadecenoic acid into an unusual *bis*-methylene-interrupted diene instead of native methylene-interrupted dienoic FAs with double bonds at C6 and C9 of the acyl chain. The mass-spectrum of this FA is presented in Figure 2b. It is typical in that it has prominent ions at *m*/*z* = 92, 108, 151 and 164, which are all fragments of the pyridine ring (https://www.lipidmaps.org/resources/lipidweb/lipidweb_html/ms/pyrcarb.htm (accessed on 30 November 2022)). The truly distinctive feature of this molecule is the ion at *m*/*z* = 232, representing a cleavage at the center of the *bis*-methylene-interrupted double bond system. In addition, gaps of 12 amu between *m*/*z* = 206 and 218 as well as *m*/*z* = 260 and 272, together with corresponding gaps of 40 amu between *m*/*z* = 178 and 218 and *m*/*z* = 232 and 272 (see also Table 2), pointed to the location of the double bonds at positions Δ^6^ and Δ^10^.

## 4. Discussion

FADs are highly specific towards the length of their acyl substrates, as well as to the position and geometric configuration of the newly introduced *cis* double bonds [8]. The counting mode (from a carboxyl (Δ) or a methyl (ω) terminus) of some FADs is a long-standing question with yet no clear answer [14]. Monounsaturated oleic acid has a single double bond that may be equally assigned to Δ^9^ or ω^9^ positions. The counting mode of plant-type soluble Acyl-Carrier-Protein (ACP) desaturases was experimentally determined relative to the carboxyl end of the FA [15,16] (Δ positioning). Instead, integral acyl-lipid desaturases may introduce double bonds between the specific carbon atoms counting from the carboxyl (Δ) or methyl (ω) terminus of the FA chain. In addition, a subsequent double bond may be introduced to three carbons (3C) from a pre-existing double bond towards a carboxyl terminus (front-end desaturases) or methyl terminus (methyl-end desaturases) [17].

Higher plant Δ6-FAD have been assigned to a class of front-end desaturases [1,2] that catalyze the introduction of a double bond between the preexisting Δ^9^ double bond and the carboxyl-group. The counting mode of cyanobacterial acyl-lipid Δ6-desaturases was not studied before. Cyanobacterial Δ6-FADs have been cloned from *Synechocystis* sp. strain PCC 6803 [5] and *Arthrospira* (*Spirulina*) *platensis* [6] and expressed in *Escherichia coli* [18], which has no FADs, and in yeast [19], which has one Δ9-FAD, and is capable of synthesizing only monoenoic Δ^9^-FAs. However, those investigations applied the standard techniques of FA analysis and did not focus on the analysis of minor fractions of FAs. Therefore, the appearance of monoenoic Δ^6^ desaturated FAs was, probably, masked by the major FA fractions in the presented chromatograms.

The experimental data on the appearance of Δ^6^ desaturated monoenoic FAs in *Synechocystis* sp. PCC 6803 (and in *Spirulina*/*Arthrospira platensis*) is absent. The accepted scheme of desaturation events in cyanobacteria implies that Δ6-FAD acts as a front-end desaturase, which uses Δ^9^ monoenoic or Δ^9,12^ dienoic FAs as substrates [20,21]. It points to the conclusion, that, at least, in these cyanobacterial strains, Δ6-FAD may prefer monoenes and dienes as substrates.

Previously, we characterized cyanobacterial Δ12-FADs as methyl-end desaturases that “count” 3C toward the methyl end from the pre-existing double bond in the monoenoic precursors irrespective of a FA chain length: monounsaturated odd-chain 17:1Δ^10^ was converted into 17:2Δ^10,13^, whereas even-chain 18:1Δ^11^ was converted into 18:2Δ^11,14^ [9].

If Δ6-FAD would act as a front-end desaturase, which requires the pre-existing double bond(s) for the correct “counting”, we should not observe any monoenoic FAs desaturated at position Δ^6^. Instead, *S. elongatus* cells, which expressed Δ6-FAD, produced unusual FAs monounsaturated at position Δ^6^ (14:1Δ^6^, 16:1Δ^6^, and 18:1Δ^6^), as well as dienoic 14:2Δ^6,9^, 16:2Δ^6,9^ and 18:2Δ^6,9^ (Figure 1, Table 1). The appearance of Δ^6^ monounsaturated FAs of different lengths demonstrates the ability of the acyl-lipid Δ^6^-desaturase to introduce the first double bond into the unsaturated substrates.

The appearance of Δ^6,9^ dienes still leaves a question of whether Δ6-FAD counts from the carboxyl terminus, irrespective of the absence or presence of double bond(s) or from the pre-existing double bond. If Δ6-FAD counts 3C toward the *C*-terminus from a pre-existing double bond, then *cis*-10-heptadecenoic acid (17:1Δ^10^) should be converted into *cis*-7,10-heptadecadienoic acid, 17:2Δ^7,10^. However, exogenously supplied 17:1Δ^10^ was converted into *cis*-6,10-heptadecadienoic acid, 17:2Δ^6,10^ (Figure 2b). This indicated that the cyanobacterial Δ6-FAD counts from the carboxyl end, irrespective of the absence/presence of a double bond in an FA chain.

## Figures and Tables

**Table 1:** FA composition (%) of *S. elongatus* (WT) and its trasformant that expressed the *desD* gene.

FA	WT	*desD*
14:0	0.6	1.3
**14:1Δ** **^6^**	nd	**0** **.** **5**
14:1Δ^9^	1.1	1.1
16:0	55.6	48.7
**16:1Δ** **^6^**	nd	**9** **.** **7**
16:1Δ^9^	35.8	21.0
**16:2Δ** **^6,9^**	nd	**7** **.** **4**
18:0	1.4	3.5
**18:1Δ** **^6^**	nd	**1** **.** **5**
18:1Δ^9^	4.1	3.5
18:1Δ^9,11^	1.4	0.4
**18:2Δ** **^6,9^**	nd	**1** **.** **4**

The appearance of unusual monoenoic and dienoic acids, supposedly, and the products of the activity of the expressed *desD* gene are indicated in bold. The individual peaks were identified with the program MSD Chem Station and NIST spectrum library. nd—not determined. All experiments were repeated at least 3 times. The deviation of values was within 0.1–0.5%.

**Table 2:** Chromatographic features of FAMEs and mass-spectrometry features of 3-pyridilcarbinyl derivatives of FAs from cell *S. elongatus* PCC 7942 transformed with the *desD* (*sll0262*) gene and grown in presence of potassium salt of *cis*-10-heptadecenoic acid (17:1Δ^10^). RT—retention time; RRT—relative retention time to C18:0; ECN—equivalent carbon number; M^+^—molecular ion peak; amu—atomic mass unit(s).

FA	RT min	RRT18:0	M^+^	12 amu Gap	40 amu Gap
14:0	10.276	0.602			
14:1Δ^6^	10.926	0.640	317	206–218	178–218
14:1Δ^9^	11.136	0.653	317	248–260	234–274
14:2Δ^6,9^	12.32	0.722	315	206–218, 246–258	178–218, 218–258
16:0	12.96	0.759			
16:1Δ^6^	13.821	0.810	345	206–218	192–232
16:1Δ^9^	14.006	0.821	345	248–260	234–274
17:1Δ^10^	14.758	0.865	359	262–274	248–288
16:2Δ^6,9^	15.276	0.895	343	206–218, 246–258	232–272, 192–232
17:2Δ^6,10^	16.956	0.994	357	206–218, 260–272	178–218, 232–272
18:0	17.064	1.000			
18:1Δ^6^	18.159	1.064	373	220–232	206–246
18:1Δ^9^	18.264	1.070	373	248–260	234–274
18:1Δ^11^	18.465	1.082	373	276–288	248–288
18:2Δ^6,9^	19.615	1.149	371	206–218, 246–258	178–218, 232–272

## Data Availability

Not applicable.
